# La pubalgie du sportif: mise au point à propos d'une étude rétrospective de 128 joueurs

**DOI:** 10.11604/pamj.2015.22.288.8136

**Published:** 2015-11-24

**Authors:** Ammar Mahmoudi, Samia Frioui, Sonia Jemni, Faycel Khachnaoui, Younes Dahmene

**Affiliations:** 1 Service de Chirurgie Générale et Digestive, CHU Fattouma Bourguiba de Monastir, Tunisie; 2 Service de Médecine Physique Réadaptation Fonctionnelle, CHU Sahloul, Route de Ceinture 4011 H Sousse, Tunisie; 3 Clinique Les Oliviers, Boulevard 7 Novembre, 4051, Sousse, Tunisie

**Keywords:** Pubalgie, sportif, chirurgie, prévention, rééducation, intervention de Bassini, Pubalgia, athlete, surgery, prevention, re-education, Bassini repair

## Abstract

La pubalgie du sportif de haut niveau représente une entité nosologique à part entière, tant en raison du mécanisme à l'origine de la pathologie que des lésions objectivables au niveau de la paroi abdominale. C'est un syndrome douloureux de la région inguino-pubienne qui touche particulièrement les footballeurs. Son étiologie est attribuée à la répétition de mouvements des membres inférieurs et du tronc associant rotation et adduction forcées. Son incidence est nettement plus élevée chez les hommes. Après avoir écarté une pathologie d'organe, le patient devrait bénéficier tout au début de son traitement conservateur d'une IRM afin d'effectuer un bilan lésionnel complet qui, selon les situations, permet d’écourter le traitement conservateur et de proposer un traitement chirurgical optimal. Le traitement doit être résolument conservateur pendant 3 mois. La rééducation est le plus souvent le traitement de première intention. Les patients présentant une persistance des symptômes sont candidats à la chirurgie. L'intervention de Nesovic est le traitement de choix chez les sportifs de haut niveau et qui permet, dans la très grande majorité des cas, une reprise sans aucune limitation de l'activité sportive antérieure. La technique de Bassini semble être moins lourde que celle de Nesovic, puisqu'elle est moins invasive. Une prise en charge multidisciplinaire centrée sur l'athlète avant et après l'intervention permet un retour aux activités physiques après quelques mois. Nous rapportons l'expérience de la prise en charge de 128 joueurs opérés selon la technique de Bassini et nous comparons nos résultats avec celles de la littérature.

## Introduction

La pubalgie se définit comme un syndrome douloureux de la région inguinopubienne. Elle est très fréquente chez le footballeur, lorsqu'il existe un surmenage sportif avec microtraumatismes répétés ou de fortes contraintes imposées lors de la pratique de ce sport sur les muscles adducteurs et le pubis. Elle touche principalement des footballeurs et presque exclusivement des athlètes masculins. Elle se rencontre dans d'autres sports, notamment le rugby, le tennis, le handball ou le hockey sur glace. Son installation peut être aiguë après un traumatisme franc, mais elle est le plus souvent d'apparition insidieuse en raison de microtraumatismes répétés. Les pubalgies posent un problème diagnostique et thérapeutique, d'autant plus important qu'elles vont éloigner du terrain les joueurs pour plusieurs semaines ou mois. Devant une douleur de la région pubienne, après avoir éliminé une pathologie infectieuse ou d'organe, on retient le plus souvent dans un premier temps, le diagnostic de pathologie de surcharge musculo-tendineuse. Le patient est alors mis au bénéfice d'un traitement conservateur. La persistance de la symptomatologie après un traitement conservateur bien conduit donne la place au traitement chirurgical. Plusieurs techniques ont été utilisées, la plus célèbre est celle de Nesovic. Le choix d'une technique chirurgicale adaptée est crucial et toujours en débat depuis Nesovic [1]. Nous rapportons notre expérience de la prise en charge de 128 sportifs de haut niveau opérés selon la technique de Bassini.

## Méthodes

Notre étude est rétrospective portant sur 128 joueurs opérés par le même chirurgien, au CHU Sahloul et la clinique des oliviers de Sousse en Tunisie, pour pubalgie selon la technique de Bassini entre 2007 et 2014. Tous les joueurs ayant présenté un syndrome douloureux évocateur de pubalgie ont été inclus dans cette étude. Nous n'avons pas inclut les joueurs porteurs d'une défaillance pariétale sans syndrome douloureux associé et les joueurs présentant un syndrome de ténopathie du moyen adducteur isolé. Les données épidémiologiques ont été recueillies à partir des dossiers médicaux. Nos résultats ont été comparés à ceux de la littérature au niveau épidémiologique, clinique, para-clinique et thérapeutique.

## Résultats

L’âge moyen de nos joueurs était de 25,8 ans avec des extrêmes allant de 16 à 46 ans. Cent douze joueurs avaient un âge entre 16 et 29 ans (87,5%). On relève une très nette prédominance masculine avec 124 cas (96,9%). Les sports pratiqués étaient les sports collectifs avec en premier lieu le football dans 119 cas (92,9%), le handball dans sept cas (5,5%), le basketball dans un cas (0,8%) et le volleyball dans un cas (0,8%). Les postes de jeu des footballeurs étaient: défenseur dans 36 cas (30,25%), milieu dans 50 cas (42%), attaquant dans 32 cas (26,9%) et gardien dans un cas (0,85%). Le nombre moyen d'heures de jeu était de huit heures par semaine avec des extrêmes allant de quatre à dix heures. Des antécédents de pubalgie opérée étaient retrouvés dans sept cas (5,5%), de chirurgie sur le genou dans cinq cas (3,9%), de hernie inguinale opérée dans un cas (0,8%). Les joueurs n'avaient pas d'antécédents médicaux notables. Le tabagisme a été retrouvé dans 14 cas (10,9%). Le début de la douleur était progressif dans 114 cas (89,1%). La douleur apparaissait pendant l'effort dans 72 cas (56,2%). Son siège était sus-pubien chez 122 patients (95,3%) et sous-pubien chez six malades (4,7%). Elle était responsable de l'arrêt de l'activité sportive chez 53 joueurs (41,4%). Le signe de Malgaigne était présent chez 88 patients (68,8%). Une douleur à l'insertion pubienne des muscles grands droits était notée chez 23 patients (17,9%). Une pointe de hernie inguinale était notée chez six patients (4,7%). L'examen de la symphyse pubienne avait montré une douleur à la pression dans 74 cas (57,81%) sans mobilité clinique anormale. Une douleur à la palpation des tendons adducteurs à leur insertion pubienne était retrouvée chez 59 joueurs (46,1%). L'abduction forcée et l'adduction contrariée de la hanche étaient douloureuses chez ces mêmes patients. La radiographie du bassin de face était pratiquée chez 27 joueurs (21,1%) et elle a été sans particularité. l'IRM a été réalisée chez 34 patients (26,56%) et a permis de retenir le diagnostic de pubalgie. La numération et formule sanguine pratiquée chez tous les sportifs avait montré une hyperleucocytose dans 13 cas (10,2%) et était normale dans 115 cas (89,8%). Un traitement conservateur était prescrit chez tous les patients. Ce traitement était à base d'antalgiques et d'anti-inflammatoires avec un repos strict de un à trois mois. Après le repos sportif, des exercices d’étirement des ischio-jambiers et des adducteurs avec des exercices de renforcement de la sangle abdominale ont été pratiqués pendant une durée d'au moins trois mois. Le traitement chirurgical était indiqué devant soit un échec du traitement médical avec persistance de la douleur et absence de reprise de l'activité sportive soit une récidive de la douleur dès la reprise de l'activité sportive soit une défaillance nette pariéto-abdominale chez un sportif motivé.

Une rachianesthésie a été pratiquée chez 71 joueurs (55,5%) et une anesthésie générale chez le reste des sportifs (44,5%). Tous les joueurs étaient opérés par voie antérieure qui était bilatérale dans 70 cas (54,7%), unilatérale droite dans 26 cas (20,3%) et unilatérale gauche dans 32 cas (25%). Une défaillance du plancher inguinal était notée dans 117 cas (91,4%). Une atrophie et une rétraction du tendon conjoint étaient retrouvées chez 118 patients (92,2%). Une pointe de hernie inguinale était constatée chez 14 joueurs (10,9%). Tous les patients avaient bénéficié d'une cure à la Bassini. Une aponévrotomie de décharge du tendon conjoint était pratiquée chez 121 patients (94,5%). Une ténotomie percutanée du tendon du moyen adducteur était réalisée chez 103 malades (80,5%). Le sac herniaire était réséqué dans les 14 cas de hernies constatées. La durée d'hospitalisation des patients était de 48 heures dont 24 heures en post-opératoire. Les suites opératoires étaient simples chez 127 joueurs. Une ténotomie percutanée du moyen adducteur était compliquée d'un hématome sous-cutané (0,8%). Le délai, du travail musculaire spécifique après l'intervention était précisé dans 95 cas (74,21%). Il était de un mois pour 62 patients et de 3 mois pour les 33 autres. La durée moyenne de la rééducation était de 4 semaines avec des extrêmes de 3 et 8 semaines. L'activité sportive a été reprise par 107 joueurs (83,59%), dont 98 au même niveau et 9 à un niveau inférieur. Quatre joueurs avaient arrêté la pratique du sport (3,12%). Pour le reste l’évolution n'a pas été précisée. La douleur avait complètement disparu chez 92 (71,87%) sportifs. Elle avait récidivé à l'effort chez 25 joueurs (19,53%). Un seul sportif avait gardé une douleur au cours des activités de la vie quotidienne (0,8%).

## Discussion

### Etude épidémiologique

Découverte par Spinelli, il y a près d'un siècle, le diagnostic et la prise en charge de la pubalgie demeurent la source d'un dilemme toujours aussi vivace [2]. En 1966, Cabot [3] a rapporté 202 cas de pubalgie chez 42000 footballeurs, soit 0,5% durant une période de 30 ans. Une étude Yougoslave faite en 1977 chez 1475 footballeurs a trouvé 92 (6,24%) douleurs de l'aine [3]. En 1980 Peterson et Renstrom ont constaté, dans leurs études prospectives, que 5% des plaintes chez les footballeurs étaient au niveau de l'aine [4]. Gibbon [5] dans une enquête (n = 2335) évalue la morbidité de la pubalgie dans 92 clubs professionnels européens. Il montre combien le football de haut niveau est encore frappé par la pubalgie. Près d'un joueur sur quatre est touché pendant la saison étudiée, 25 à 32% de ces joueurs récidivent pendant la même saison. La pubalgie représente beaucoup plus que les 5% de la pathologie du footballeur présenté par Gilmore [6]. La pubalgie atteint le sportif jeune en pleine activité avec un maximum de fréquence entre 20 et 30 ans [7]. Christel a révélé un âge de survenue entre 18 et 40 ans avec une moyenne de 25 ans [8]. Arezki a révélé un maximum de fréquence entre 22 et 24 ans [9] et Taylor un âge moyen de 24 ans avec des extrêmes de 13 et 33 ans [10]. La moyenne d’âge de notre population était de 25,8 ans avec des extrêmes de 16 et 46 ans et un maximum de fréquence se situant entre 20 et 24 ans. Une nette prédominance masculine a été notée dans toutes les études. Christel, dans sa recherche, a présenté 2 femmes sur les 65 cas opérés [11]. Adrian [3] et Volpi [12] ont présenté chacun une femme dans son étude. La majorité de nos patients (96,9%) étaient de sexe masculin.

### Etude clinique

Le diagnostic d'une pubalgie secondaire à un déséquilibre mécanique siégeant au niveau musculaire entre les adducteurs d'une part trop puissants, et les muscles larges insuffisants de la paroi antérolatérale de l'abdomen d'autre part, se base essentiellement sur l'anamnèse et l'examen clinique. Les examens complémentaires sont peu utiles et permettent essentiellement dans ce contexte d’écarter d'autres diagnostics. L'anamnèse révèle tout d'abord une activité sportive régulière et souvent intensive qui concerne en très grande majorité les footballeurs (environ 70%) [11] mais qui peut néanmoins concerner des coureurs de fond, des escrimeurs, des joueurs de hockey ou de tennis ainsi que des nageurs [8]. La douleur, aggravée lors de l'effort et des man'uvres de Valsalva (toux, défécation), débute le plus souvent de manière progressive; cependant son apparition peut être brutale, survenant lors d'un mouvement d'abduction forcée en rotation externe [8]. Elle est de localisation sus et / ou infra-pubienne, la plupart du temps unilatérale mais parfois en barre abdominale et irradie vers le pubis et les testicules. En cas d'association à des algies des adducteurs, c'est la douleur sus-pubienne qui prédomine. L'examen clinique fait apparaître de manière inconstante des signes aspécifiques qui sont l'apparition de douleurs lors de la contraction isométrique tant de la musculature abdominale que des adducteurs. On doit examiner les insertions tendineuses des grands droits de l'abdomen, des adducteurs des cuisses et il faut examiner la personne debout, à la recherche d'une bascule du bassin ou d'une hyperlordose lombaire. L’élément positif qu'il faut absolument rechercher et qui est particulièrement marqué chez des patients longilignes ayant une attitude en hyperlordose avec des ischio-jambiers et des adducteurs musclés et courts est le déséquilibre de la sangle abdominale en confrontant la puissance du grand droit habituellement très développé, à celui des muscles obliques souvent insuffisants. Ceci se traduit par une déhiscence pariétale située entre le tendon conjoint et l'arcade crurale mise en évidence par le signe de Malgaigne: en hyperlordose on observe l'apparition d'une voussure oblongue entre l'arcade crurale et le bord inférieur des obliques plus marquée à la toux. Ce signe a été retrouvé chez 68,8% de nos joueurs. Le diagnostic différentiel d'une pubalgie inclut les pathologies ostéo-articulaires (arthropathie de la symphyse, instabilité de la symphyse, fracture d'une branche ilio ou ischio-pubienne, atteinte de l'articulation coxo-fémorale); les pathologies osseuses infectieuses (ostéite du pubis); les atteintes du système génito-urinaire (infections urinaires banales, prostatite chez l'homme et infections de la sphère gynécologique); les pathologies musculo-tendineuses (élongations/ déchirure des adducteurs de la hanche); les pathologies de la paroi abdominale (hernies inguinales, déhiscence ou déchirure partielle des muscles de l'abdomen); les néoplasies; les douleurs référées (de pathologies rachidiennes telles des hernies discales au niveau dorsolombaire).

### Les examens complémentaires

Les examens complémentaires doivent être ciblés afin d'exclure certaines pathologies d'organes lorsque l'anamnèse et l'examen clinique sont évocateurs et permettent dans certains cas d'instaurer un traitement étiologique. Un bilan biologique peut être effectué afin de rechercher un syndrome inflammatoire lorsque l'on évoque une ostéomyélite de la symphyse pubienne. Il peut également être utile pour exclure une atteinte infectieuse du système génito-urinaire. La radiographie standard centrée sur le pubis n'est indiquée que lorsque l'on suspecte cliniquement une fracture pathologique d'une branche ilio ou ischio-pubienne ou une fracture par arrachement au lieu d'une insertion tendineuse. La scintigraphie osseuse n'a un intérêt qu'en complément de la radiologie standard, sa sensibilité étant meilleure et les modifications scintigraphiques plus précoces. En cas de pubalgie avec une radiographie normale, une scintigraphie osseuse montrant une hypercaptation au niveau de la symphyse peut être diagnostiquée sous réserve de son manque de spécificité. En effet, toutes les lésions osseuses qu'elles soient traumatiques, tumorales ou infectieuses entraînent une hyperfixation. Le scanner ne semble apporter aucun élément supplémentaire par rapport aux radiographies standards. L'imagerie par résonance magnétique (IRM) en tant qu’élément diagnostique de la pubalgie du sportif a fait l'objet de plusieurs études [11]. C'est la seule technique d'imagerie capable d’étudier simultanément l'os des branches pubiennes, le fibrocartilage de la symphyse, les insertions tendineuses des adducteurs et des muscles larges de l'abdomen sur le pubis, ainsi que les muscles et cela dans tous les plans de l'espace. Dans le contexte de la pubalgie du sportif, l'IRM est l'examen de choix. Elle permet en effet d'effectuer un bilan lésionnel complet lors de douleurs chroniques d’étiologie peu claire et ne répondant que mal au traitement conservateur. Pour certains auteurs [11], l'IRM permet d'affiner la stratégie chirurgicale en identifiant, lorsqu'elle est présente, une enthésopathie des adducteurs associée à l'insuffisance de la paroi abdominale. L’échographie n'a d'indication que si l'on suspecte une hernie inguinale ou crurale. La mise en évidence d'une pathologie herniaire pose dans le contexte d'une pubalgie résistante au traitement conservateur et en l'absence de toute autre étiologie l'indication à un traitement chirurgical.

### Le traitement

Le traitement de la pubalgie est avant tout médical. Il associe un repos sportif complet, une physiothérapie antalgique puis une rééducation avec renforcement musculaire, ainsi qu'une médication appropriée. Le traitement chirurgical est envisagé secondairement et en cas d'insuffisance pariéto-abdominale, et ce après échec d'un traitement médical bien conduit [9]. L'essentiel des efforts doit porter sur la prévention des pubalgies, avec adaptation de l'activité physique du sportif, diminution ou élimination des facteurs favorisants.

### La prevention

La prévention de la pubalgie est essentielle. Elle consiste en un entretien de la musculature abdominale par la pratique d'une alternance de stretching et de musculation. On distingue deux types de prévention: la prévention primaire où on veillera surtout à doser les entraînements en quantité et en intensité. Bien souvent les syndromes pubalgiques apparaissent chez des sujets jeunes ayant augmenté brutalement leurs quantités d'entraînement ou chez des athlètes, arrêtés pour blessure pendant plusieurs semaines, lors de la reprise. L'entraîneur devra veiller au matériel utilisé, par exemple en football la dimension des crampons qui devra toujours être adaptée à la qualité du sol et il faudra toujours veiller à utiliser des chaussures évitant les blocages sur sols synthétiques. On déconseillera enfin chez le jeune footballeur, des ballons trop lourds, usagés, facteurs aggravant des microtraumatismes; la prévention secondaire concerne plus des facteurs intrinsèques selon la présence d'une hyperlordose (travail des abdominaux et des spinaux en verrouillage), d'un déséquilibre quadriceps-ischiojambiers (travail analytique et vérification par isocinétisme), d'un rétracté hypertonique nécessitant des assouplissements.

### Le traitement conservateur

Les données de la littérature ne permettent pas de mettre en évidence de consensus concernant la durée du traitement conservateur des pubalgies du sportif. Il va de 2 à 4 semaines pour certains auteurs [6], à 3 mois pour la plupart d'entre eux [7] et peut même aller jusqu’à 6 mois. Le traitement doit être adapté en fonction de différents critères: la ou les formes anatomo-cliniques, l’âge et les motivations du patient, le niveau sportif, le type de handicap, et l'intensité de la douleur. Il associe un repos sportif complet suffisamment prolongé pour permettre la consolidation des éléments tendino-musculo-aponévrotiques lésés, une physiothérapie d'abord antalgique puis de rééducation-renforcement musculaire ainsi qu'une médication appropriée à base d'anti-inflammatoires non stéroïdiens [7] voire même de stéroïdes per os. Dans les cas rebelles, des infiltrations peuvent être adjointes au traitement précédant. Selon la littérature, le traitement médical permet d'obtenir la guérison dans environ 80% des cas [8]. Lors de l’échec d'un traitement conservateur de longue durée bien conduit, l'indication opératoire doit être alors prise en considération.

### Le traitement chirurgical

Certains auteurs s'accordent à penser que le traitement chirurgical ne doit pas être proposé d'emblée, en première intention, mais toujours après un traitement médical [6, 8]. Le traitement chirurgical de la pubalgie a fait l'objet de nombreux travaux depuis Nésovic il y a plus de 30 ans sans que ce traitement fasse l'objet d'un réel consensus. L'indication à une prise en charge chirurgicale doit être réservée aux patients qui n'ont pas présenté d'amélioration clinique évidente après avoir bénéficié d'un traitement conservateur bien conduit d'au moins 3 mois et est réservée aux étiologies pariéto-abdominales [6, 11]. La pubalgie apparaît pour beaucoup d'auteurs [1, 8], comme un déséquilibre mécanique au niveau musculaire qui peut être corrigée chirurgicalement de deux façons: soit par une détente des muscles adducteurs, ceux-ci étant considérés comme trop forts, soit par une remise en tension des muscles larges de l'abdomen [8, 11]. La plus célèbre des interventions stabilisatrices du pubis est celle pratiquée par Nesovic [9], mais les autres techniques basées sur la réparation du canal inguinal empruntées aux traitements des hernies tels que la technique de Bassini, la technique de Shouldice, le procédé de Lichtenstein, et la laparoscopie pré-péritonéale sont aussi utilisées. Nesovic a décrit en 1984, une intervention qui réalise un rééquilibrage par plastie abdominale des forces qui entrent en jeu au niveau de la symphyse pubienne [1]. L'intervention de Bassini comporte une dissection avec incision de l'aponévrose du grand oblique, une mobilisation du cordon et une résection du crémaster. La réparation se fait par des points de suture abaissant le tendon conjoint à l'arcade crurale en arrière du cordon. L'aponévrose du grand oblique est suturée en avant du cordon. Cette intervention ne nécessite pas une résection nerveuse, ni drainage. C'est la technique la plus ancienne. Elle s'effectue sous anesthésie générale ou rachi-anesthésie. En 2003, Van Der Donckt [13] rapporte 41 cas de pubalgie chez des sportifs opérés selon la technique de Bassini, dont 14 bilatéraux, associés à une ténotomie du moyen adducteur. Trente-six des patients reviennent au même niveau. Le délai de reprise est de 6,9 mois (6 à 15). En 2001, Ëkstrand [14] rapporte 66 cas de pubalgie chez des joueurs de football opérés dont 17 selon la technique de Bassini. Il note 87% de reprise entre 6 et 8 semaines. En 1991, Poglase [15] rapporte 64 cas de pubalgies opérées chez des footballeurs Australiens, selon la technique de Bassini modifiée avec pose de patchs. Il décrit 93,8% de bons résultats. Dans notre série, tous les patients ont été opérés selon la technique de Bassini avec aponévrotomie de décharge du tendon conjoint. L'intervention était bilatérale chez les sujets ayant une déficience pariétale abdominale objective de deux côtés. Cette technique a été également utilisée par Christel [16] et Adrian [3]. La détente des muscles adducteurs peut être réalisée soit par ténotomie percutanée, essentiellement du moyen adducteur, soit par abord chirurgical direct excisant les lésions fibro-cicatricielles siégeant au niveau de l'insertion sur l'ilion des adducteurs. Cette tactique chirurgicale, est reléguée au second plan car elle est, d'une part, considérée comme trop délabrante pour être proposée à des sportifs de haut niveau [8, 10] et, d'autre part, doit être réservée aux formes de pubalgies secondaires à une tendinopathie «pure» des adducteurs, c'est-à-dire sans signe permettant d’évoquer une insuffisance de la paroi abdominale. Beaucoup d'auteurs accordent une place prépondérante au rôle joué par les adducteurs et proposent une technique qui combine un rééquilibrage de la sangle abdominale et une ténotomie percutanée des adducteurs [16]. Christel [11] associe la ténotomie du moyen adducteur à l'intervention de Nesovic dans 20% des cas, Meyers [17] dans 23% des cas. Dans notre série la ténotomie percutanée du moyen adducteur du côté de la symptomatologie douloureuse a été associée à la technique de Bassini dans 80,5% des cas. De nombreux travaux confirment les bons résultats de la chirurgie dans les formes rebelles de pubalgie. Dans une étude prospective, Van Der Donckt [13] propose à 41 footballeurs un traitement chirurgical (27 unilatérales, 14 bilatérales) après échec d'une thérapeutique classique conservatrice, associant la méthode de Bassini à une ténotomie percutanée du long adducteur. La reprise du sport se fait en moyenne à 7 mois (6 à 15 mois). La majorité (90%) retrouve le même niveau de compétition. La voie d'abord antérieure est de loin la plus utilisée. Elle est la plus simple et la seule faisable sous anesthésie locale. Dans notre série, tous les patients ont été opérés par voie antérieure bilatérale dans 54,7% des cas, unilatérale droite dans 20,3% des cas, et unilatérale gauche dans 25% des cas. La voie postérieure est utilisée en cas de chirurgie laparoscopique. La voie pré- péritonéale traverse la paroi abdominale, soit par une incision latérale supra- inguinale, soit par une incision médiane sous ombilicale, ce qui permet le traitement en un temps des deux côtés. Pour les constatations opératoires, la lésion la plus fréquente et la plus fréquemment retrouvée par les auteurs [6, 17], ainsi que dans notre série, est la déhiscence du plancher inguinal généralement associée à une atrophie et une rétraction du tendon conjoint. Une défaillance du plancher inguinal a été retrouvée dans 48% des cas et 61% des cas rétrospectivement par Meyers [17] et Adrian [3]. Dans notre série la défaillance du plancher inguinal a été retrouvée dans 91,4% des cas. Elle a été toujours associée à une atrophie et une rétraction du tendon conjoint. Une aponévrotomie de décharge a été pratiquée chez tous ces patients. Taylor et coll [10] ont trouvé une hernie inguinale dans huit cas sur neuf. Dans notre série, une pointe de hernie inguinale a été retrouvée chez 14 patients dont huit en per-opératoire. Après une laparoscopie ou une intervention de Bassini, la durée d'hospitalisation moyenne était de 24 heures, ce qui a été le cas dans notre série. En revanche, après une intervention de Nesovic, il est recommandé au patient de rester au lit avec un oreiller sous les genoux pour soulager les sutures et positionner les hanches en flexion. Les drains sont enlevés à la 48^ème^ heure. Le lever est autorisé au 8^ème^ jour [11]. Les complications post-opératoires générales sont représentées essentiellement par les complications thromboemboliques, les rétentions et l'infection urinaire, alors que les complications locales sont représentées essentiellement par les hématomes sous-cutanés, les névralgies, les douleurs récurrentes et l'infection de la paroi. Dans une étude récente portant sur 100 cas de pubalgie opérée [18], cinq cas d'hématome ont été rapportés. Dans notre série un hématome a compliqué une ténotomie percutanée. La plupart des chirurgiens s'accordent pour ne débuter la rééducation qu'après 2 à 4 semaines de repos post-opératoire [8]. Elle est basée dans un premier temps sur un travail musculaire isométrique qui débute vers le 15^ème^ jour post-opératoire pour ensuite devenir isotonique à partir de la 6^ème^ semaine. La reprise de l'activité sportive survient entre le 60^ème^ et le 90^ème^ jour post-opératoire et se doit d’être progressive [8, 16]. Gilmore [6] propose un programme de reprise sportive plus précoce, l'entraînement est repris dans le courant de la 5^ème^ semaine. En fait c'est la clinique qui doit guider le thérapeute. Une bonne souplesse des adducteurs, une récupération satisfaisante de la sangle abdominale, un test de contraction résistée des adducteurs négatif permettent la reprise de la pratique sportive. Dans notre série 83,59% des sportifs ont pu reprendre leur activité sportive dont 91,58% au même niveau et 8,42% à un niveau inférieur.

## Conclusion

La pubalgie du sportif est actuellement une entité bien connue. Sa prise en charge chez le sportif est un processus long qui s’étale sur plusieurs mois. Elle nécessite une approche spécialisée, multidisciplinaire coordonnée, centrée sur l'athlète blessé. Un diagnostic précoce permet un traitement adapté et efficace. Une fois le diagnostic de pubalgie du sportif posé, le clinicien guide et évalue régulièrement la prise en charge de rééducation par un examen clinique le plus sensible, spécifique et reproductible possible. La prévention primaire et secondaire par des programmes d'exercices physiques réguliers a montré dans plusieurs études une réduction significative de l'incidence de pubalgie chez le sportif. Dans certains cas le traitement devra être chirurgical si le traitement conservateur est en échec ou si la lésion présente un potentiel de cicatrisation faible. Il ne doit pas être retardé plus de trois mois, car les résultats sont excellents aussi bien pour les techniques classiques que laparoscopiques.
